# ACK2 antibody conditioning enhances adoptive transfer of hematopoietic progenitors to study central trained immunity in mice

**DOI:** 10.3389/fimmu.2026.1735878

**Published:** 2026-03-04

**Authors:** Andrea Guiu, Paula Guerrero, María Sobén, Daniel Gozalbo, M. Luisa Gil, Alberto Yáñez

**Affiliations:** Instituto de Biotecnología y Biomedicina (BIOTECMED) and Departamento de Microbiología y Ecología, Facultat de Ciències Biològiques, Universitat de València, Burjassot, Spain

**Keywords:** ACK2, adoptive transfer, antibody, central trained immunity, c-kit, hematopoietic progenitor, neutrophils

## Abstract

Hematopoietic stem and progenitor cell (HSPC) transplantation is a cornerstone for studying hematopoiesis. However, classical conditioning regimens such as irradiation or chemotherapy induce strong inflammation, alter the bone marrow (BM) microenvironment, and severely limit the interpretation of differentiation processes. Moreover, donor HSPC engraftment efficiency in immunocompetent recipients without conditioning is usually very low. In this work, we produced and purified the monoclonal anti-c-Kit antibody ACK2 and tested its capacity to transiently deplete HSPCs in immunocompetent C57BL/6 mice. We defined the *in vivo* clearance kinetics of the ACK2 antibody from serum, identified the optimal transplantation window, and evaluated donor engraftment efficiency. Intraperitoneal injection of ACK2 induced transient HSPC depletion in the BM, with maximal depletion and complete clearance of circulating antibody at day 4 post-injection. Transplantation of donor HSPCs in ACK2-conditioned recipients at this time point resulted in significantly improved engraftment compared to PBS-treated recipients, particularly in the BM. As a proof of concept, we applied this mouse model to investigate properties of innate immune memory in HSPCs exposed to *Candida albicans in vivo*. For this, we adoptively transferred HSPCs from infected mice with a non-virulent *C. albicans* strain and assessed the functional properties of their derived neutrophils *in vivo*. We found that neutrophils derived from *C. albicans*-exposed HSPCs displayed an enhanced recruitment to the peritoneal cavity during a secondary *C. albicans* infection compared to control HSPC-derived neutrophils. In conclusion, here we describe a non-inflammatory, antibody-based conditioning method that enhances adoptive transfer of HSPCs in immunocompetent mice. Consistent with previous reports, ACK2-based conditioning alone does not enable permanent hematopoietic engraftment, but rather facilitates transient donor cell engraftment which provides a versatile methodological tool to study the biology and functional programming of exogenous HSPCs *in vivo*, including their contribution to trained immunity.

## 1 Introduction

Hematopoietic stem and progenitor cell (HSPC) transplantation has long been a fundamental tool for studying hematopoiesis and immune cell function, and it underlies therapeutic approaches in hematological malignancies and genetic disorders. Traditionally, total body irradiation or high-dose chemotherapy have been used to create space in bone marrow (BM) niches for donor HSPC engraftment. Although effective, these regimens are highly toxic, inducing systemic inflammation, tissue damage, and profound alterations in the BM microenvironment (1). These side effects severely limit the interpretation of experimental studies where the goal is to investigate differentiation and functional programming of transplanted HSPCs without confounding inflammatory signals.

In unconditioned immunocompetent mice, donor HSPCs engraft poorly due to the lack of available BM niches (2). This limitation motivated the search for gentler, non-myeloablative conditioning strategies. A major advance came with the demonstration that monoclonal antibody-mediated depletion of host HSPCs could create temporary niche availability. In particular, the anti-c-Kit antibody ACK2 was shown to deplete HSPCs and enhance donor engraftment in immunodeficient Rag2^-/-^ mice (2). ACK2 binds c-Kit (CD117), a critical receptor for stem cell factor (SCF), blocking SCF-mediated signaling required for HSPC survival (2, 3). More recently, mechanistic studies have elucidated the basis for the potency of ACK2, demonstrating that its unique antagonism of SCF-c-Kit signaling directly suppresses HSPC growth and enables depletion independently of Fc-mediated cytotoxic functions (4). However, attempts to translate ACK2 conditioning into immunocompetent recipients have yielded limited or transient effects unless combined with additional treatments such as CD47 blockade, irradiation, or toxin conjugates (5–8).

Despite these limitations, transient HSPC depletion using ACK2 has unique value as a methodological tool. It allows donor HSPCs to engraft in immunocompetent hosts without the confounding inflammation induced by irradiation or cytotoxic drugs. Thus, it represents a powerful system for studying the biology of exogenous HSPCs, their differentiation dynamics, and their functional contributions *in vivo*. Here, we developed and characterized an ACK2-based transient depletion model in C57BL/6 mice. We defined the kinetics of ACK2 antibody clearance, established the optimal transplantation window, and demonstrated improved donor HSPC engraftment.

Innate immune memory, or trained immunity, has emerged as a central concept redefining the functional potential of the innate immune system. Unlike the classical view of innate responses as transient and non-adaptive, multiple studies have demonstrated that myeloid cells and their progenitors can undergo metabolic and epigenetic reprogramming after exposure to microbial or inflammatory stimuli, resulting in enhanced responses to subsequent challenges (9, 10). This phenomenon reconciles the short lifespan of innate effector cells, such as neutrophils and monocytes, with the long-lasting nature of trained immunity, by establishing that HSPCs in the BM act as a central reservoir of innate immune memory (11–14). In this context, our group has previously shown that HSPCs from mice infected with the low-virulence *Candida albicans* strain PCA2 are reprogrammed to generate trained macrophages with heightened proinflammatory cytokine production, conferring protection against secondary infection (15). More recently, we demonstrated that HSPCs exposed *in vitro* to *C. albicans* can also give rise to trained neutrophils, which display both increased cytokine production and enhanced microbicidal capacity through elevated mitochondrial ROS generation (16).

These findings underscore the central role of HSPCs as mediators of innate immune memory and highlight the importance of experimental systems that allow *in vivo* analysis of their differentiation and functional imprinting under conditions that minimally perturb hematopoietic homeostasis. Conventional myeloablative conditioning regimens induce systemic inflammation, tissue damage, and extensive remodeling of the BM microenvironment, which can directly influence HSPC proliferation, differentiation, and epigenetic states. As a result, these approaches may introduce confounding signals that overlap with or mask the intrinsic programming associated with trained immunity. Consequently, non-inflammatory and targeted conditioning strategies are particularly advantageous when the objective is to interrogate how prior microbial exposure reprograms HSPCs and shapes the functional properties of their progeny under steady-state conditions.

As a proof of concept, we applied the ACK2-conditioned mouse model to investigate the functional programming of HSPCs exposed to *C. albicans*, and showed an improved ability of their derived neutrophils to be recruited to the peritoneal cavity during a secondary infection. Our work highlights the utility of ACK2 conditioning as a non-inflammatory, reproducible platform to study HSPC biology and trained immunity *in vivo*.

## 2 Materials and equipment

### 2.1 ACK2 antibody production

- ACK2 cell line hybridoma (Rat IgG2b, γ-chain), kindly provided by Dr. Irvin L. Weissman (Stanford University School of Medicine, California, USA), originally obtained from Dr. Nishikawa (Kyoto, Japan).
- Culture medium: RPMI 1640 1X GlutaMAX (Gibco, 61870044) supplemented with 50 U/mL penicillin/streptomycin (P/S) (Gibco, 15140122), 1X non-essential amino acids (NEAA) (Gibco, 11140050), 1X sodium pyruvate (Gibco, 11360039), 55 mM β-mercaptoethanol (Sigma, M3148), and 10%, 5% or 2.5% fetal bovine serum (FBS) (Gibco, A5256801). Note: Filter (0.22 µm) the culture medium before use.
- 50 mm and 90 mm culture dishes, and T175 flasks (Thermo Fisher 11359273, 101R20 and 159910 respectively).
- Stericup™ vacuum-driven sterile filters (0.22 µm) (Merck Millipore, S2GVU11RE).
- Neubauer chamber.
- Trypan blue solution 0.4% (Corning, 25-900-Cl).
- CO_2_ incubator (37 °C, 5% CO_2_, 95% relative humidity).
- Inverted light microscope.
- Class II biological safety cabinet.

### 2.2 ACK2 antibody concentration

- Filtered ACK2 supernatant.
- micon^®^ Ultra 15 mL centrifugal filter units, 100 kDa MWCO (Merck Millipore, UFC910024).
- Refrigerated centrifuge (Eppendorf 5430R).
- Ice bucket.

### 2.3 ACK2 antibody purification

- ACK2 ultra-concentrate.
- NAb™ Protein G Spin Column, 5 mL (Thermo Fisher, 89961).
- Pierce™ Protein G IgG Binding Buffer, pH 5.0 (Thermo Fisher, 21019).
- Pierce™ IgG Elution Buffer, pH 2.8 (Thermo Fisher, 21004).
- 1 M phosphate buffer, pH 8.4.
- pH meter.
- Zeba™ Spin Desalting Columns, 5 mL, 40K MWCO (Thermo Fisher, 89882).
- NanoDrop 2000 spectrophotometer (Thermo Fisher).
- Sterile PBS (Corning, 21-040-CM).

### 2.4 Protein electrophoresis

- Purified ACK2 antibody.
- Laemmli Sample Buffer 1X (Bio-Rad, 161-0747).
- Dithiothreitol 5% (Bio-Rad, 161-0610).
- Heating block or thermostatic water bath.
- 12% Tris-glycine NB protein gels, 10-well (NuSep, NB10-012).
- Electrophoresis chamber and power supply.
- 1X running buffer (Tris/Glicine/SDS) (Bio-Rad, 161-0732).
- Molecular weight marker: Precision Plus Protein Dual Color Standard (Bio-Rad, 161-0374).
- Quick Coomassie Stain (Neo Biotech, NB-45-00078-1L).

### 2.5 RBC-lysed bone marrow and spleen cell isolation

- Mice femur and tibia and/or spleen.
- 25G needle (BD Biosciences, 300600).
- 10 mL syringe (BD Biosciences, 309110).
- Flow cytometry buffer: PBS, 5% FBS, 2 mM EDTA (Invitrogen, AM9260G).
- 70 µm filter (Thermo Fisher, 22-363-548).
- Refrigerated centrifuge (Eppendorf 5430R).
- 1X Lysis buffer (BD Biosciences, 555899).
- 1 mL sterile syringe (BD Biosciences, 303172).

### 2.6 ACK2 antibody *in vitro* competing assay

- Filtered ACK2 supernatant and purified ACK2 antibody.
- RPMI 1640 1X GlutaMAX.
- Sterile PBS.
- RBC-lysed whole BM cells from C57BL/6 mice.
- Flow cytometry buffer and antibodies (see **Table 1**).
- LSRFortessa™ flow cytometer (BD Biosciences).

**Table 1:** Anti-mouse antibodies used for flow cytometry.

Antibody	Fluorochrome/conjugate	Clone	Dilution	Supplier	Catalog number
c-Kit (CD117)	PE	ACK2	1:50	BioLegend	135105
c-Kit (CD117)	BUV395	2B8	1:65	BD Biosciences	564011
c-Kit (CD117)	PE-Vio770	3C11	1:50	Miltenyi Biotec	130-125-226
Streptavidin	APC	–	1:50	BioLegend	405207
FcBlock (anti-CD16/CD32)	–	–	1:20	Miltenyi Biotec	130-092-575
Ly6G	BV711	1A8	1:65	BioLegend	127643
CD11b	FITC	M1/70	1:100	BioLegend	101206

### 2.7 Mice

- C57BL/6 mice (Envigo).
- DsRed.T3 transgenic mice (B6.Cg-Tg[CAG-DsRed*MST]1Nagy/J strain, The Jackson Laboratory).
- Experiments were conducted with 10- to 12-week-old mice of both sexes.

### 2.8 *In vivo* ACK2 administration, antibody clearance, and HSPC detection

- Purified ACK2 antibody.
- Syringe and 26G needle for intraperitoneal injection (BD Biosciences, 305501).
- 21G needle for peripheral blood extraction (BD Biosciences, 301156).
- Peripheral blood samples.
- RBC-lysed whole BM cells from C57BL/6 mice.
- Flow cytometry buffer and antibodies (see **Table 1**).
- LSRFortessa™ flow cytometer (BD Biosciences).

### 2.9 HSPC purification and transplantation

- BM cells from DsRed.T3 mice.
- Flow cytometry buffer and antibodies (see **Table 1**).
- Lineage Cell Depletion Kit (Miltenyi Biotec, 130-090-858).
- AutoMACS^®^ Pro Separator (Miltenyi Biotec).
- Sterile PBS.
- Syringe and 30G needle for intravenous injection (BD Biosciences, 324826).

### 2.10 *C. albicans* infection

- *C. albicans* PCA2 strain (provided by Dr. Cassone, Istituto Superiore di Sanità, Rome, Italy) (15, 17).
- *C. albicans* ATCC 26555 strain.
- Endotoxin-free YPD medium: 1% yeast extract, 2% peptone, 2% dextrose (Liofilmchem, 611005, 611004, 611601 respectively).
- Incubating Mini Shaker.
- Spectrophotometer.
- Cell Culture Grade Water (Corning, 25-055-CM).
- Neubauer chamber.
- Sterile PBS.

### 2.11 Peritoneal cavity lavage

- 70% ethanol.
- Flow cytometry buffer.
- 10 mL syringe with 25G needle.
- 15 mL Falcon tubes.
- Funnel.

### 2.12 Flow cytometry antibodies

- Antibodies used for flow cytometry are detailed in **Table 1**.

## 3 Methods

### 3.1 ACK2 antibody production

**Figure 1:**
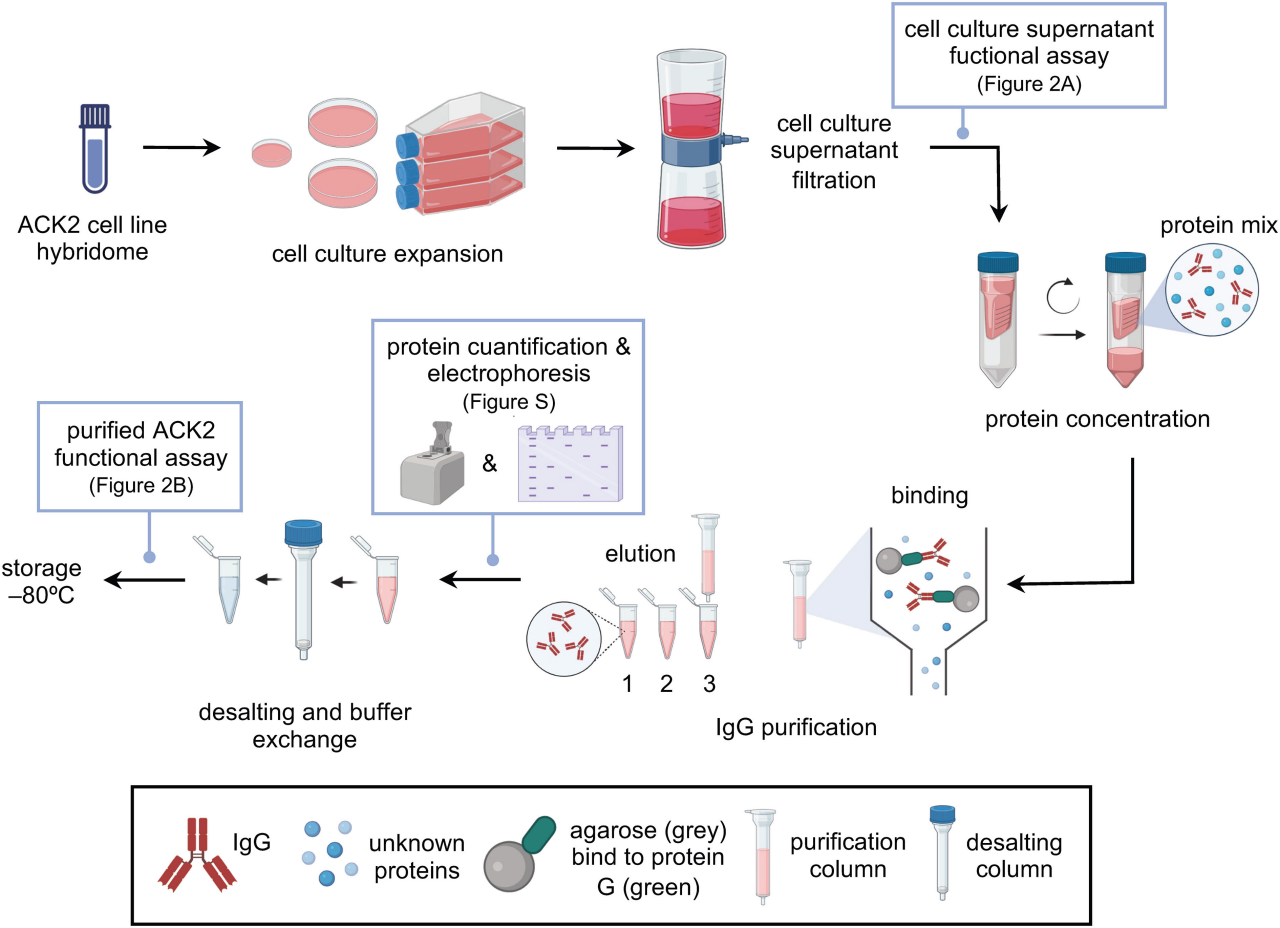
Steps for ACK2 antibody production and purification. ACK2 hybridoma vials were thawed and cultured to expand cells and accumulate antibody in the supernatant, which was subsequently filtered and stored. Once the presence of ACK2 antibody was confirmed in the culture medium, it was concentrated using centrifugal filter devices and purified by IgG affinity chromatography. Protein concentration was determined in the eluted fractions, and purity was assessed by protein electrophoresis. Selected fractions were pooled, desalted, and buffer-exchanged into PBS. Finally, antibody concentration and functionality were re-evaluated prior to experimental use. Figure created in BioRender.com. Guiu, A (2026).

**Figure 2:**
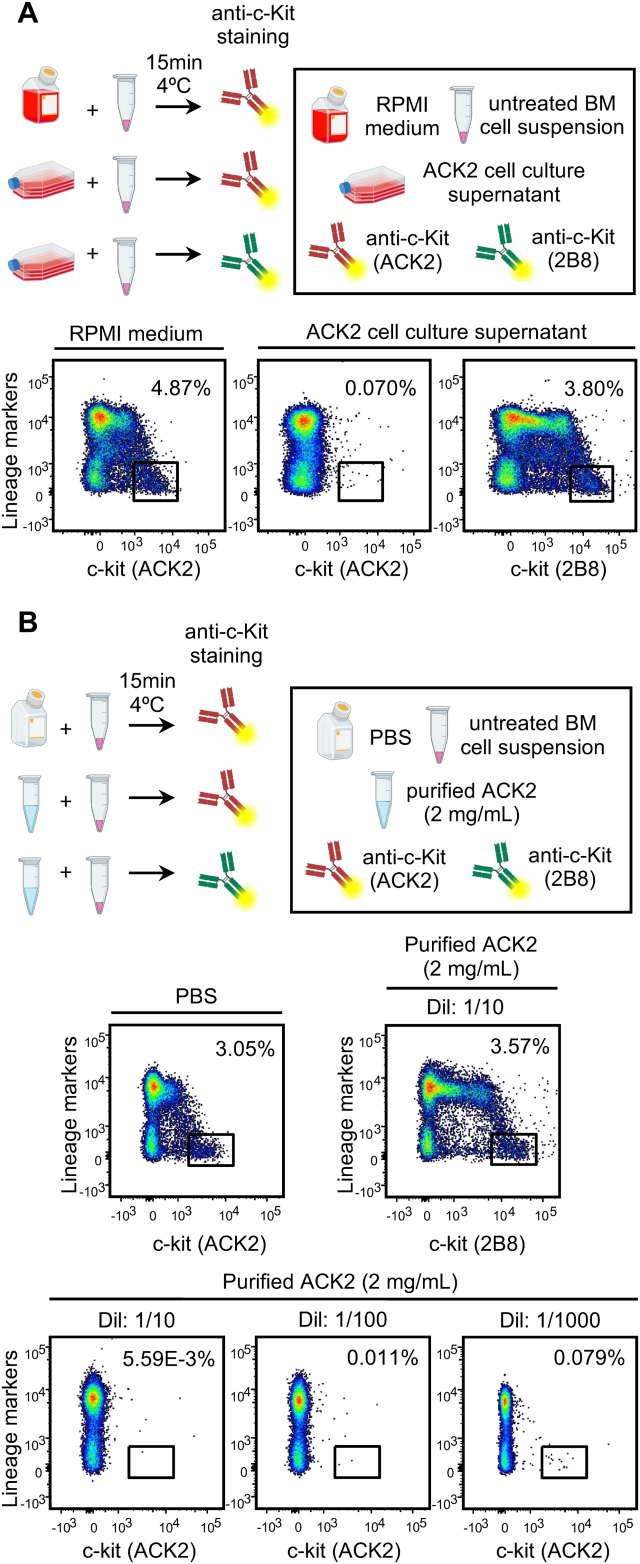
ACK2 antibody *in vitro* competing assay. **(A)** Schematic representation of the assay to detect ACK2 antibody in the culture supernatant of hybridoma cells (top). Representative dot plots show competition of supernatant-derived ACK2 with fluorescently-labeled anti-c-Kit clones ACK2 or 2B8 for binding to the Lin^–^ c-Kit^+^ population in BM samples (bottom). **(B)** Schematic representation of the assay to evaluate the functionality of purified ACK2 antibody at three dilutions (1/10, 1/100, and 1/1000) (top). Representative dot plots show competition of 1/10, 1/100 or 1/1000 dilutions of purified ACK2 antibody (2 mg/mL) with fluorescently-labeled anti-c-Kit clones ACK2 or 2B8 for binding to the Lin^–^ c-Kit^+^ BM population (bottom). Dot plots shown are representative of individual samples (n = 3) from two independent experiments with two independent ACK2 production batches. Figure partially created in BioRender.com. Guiu, **(A)** (2026).

ACK2 hybridoma cell line vials were thawed, washed, centrifuged (150 g, 5 min, 4 °C), and resuspended in ACK2 culture medium at 37 °C, 5% CO_2_, and 95% relative humidity. Cells were plated in a range of 0.3-0.5 × 10^6^ cells/mL concentrations to ensure its growth and survival in 50 mm (4mL) and 90 mm (15 mL) culture dishes, and T175 (30 mL) flasks sequentially. During expansion, the FBS concentration was gradually reduced (10%, 5%, and 2.5%) to minimize protein contamination prior to antibody purification. Cell density and viability were assessed using a Neubauer chamber and 0.4% trypan blue exclusion. Cells were passaged upon reaching 80% confluence. Once sufficiently expanded, cells were allowed to undergo slow cell death for 10–15 days to maximize antibody production. When culture viability reached ~10-20%, the medium was collected, centrifuged (150 g, 5 min, 4 °C), filtered using vacuum-driven sterile filters, and stored at -20 °C until protein concentration (**Figure 1**). The supernatants were tested for the presence of ACK2 antibody (see section 3.6, “ACK2 antibody *in vitro* competing assay”; **Figure 2A**).

### 3.2 ACK2 antibody concentration

ACK2 antibody-containing supernatants were concentrated in two rounds using centrifugal filter devices (**Figure 1**). In the first round, 12 mL of filtered supernatant were centrifuged (3000 g, 7 min, 4 °C). The retained concentrate was collected and subjected to a second round of centrifugation (3500 g, 8 min, 4 °C). ACK2 antibody-containing supernatants must be kept on ice throughout the entire concentration process. The resulting ultra-concentrate was stored at -20 °C until purification.

### 3.3 ACK2 antibody purification

ACK2 antibody was purified using an IgG affinity purification column, following the manufacturer’s protocol (**Figure 1**). Briefly, the ACK2 ultra-concentrate was diluted 1:2 in Binding Buffer (pH 5), and 10 mL were incubated (10 min, 25 °C) with end-over-end mixing and centrifuged (1000 g, 1 min, 25 °C) through the column. The non-bound sample components were subjected to another round of incubation and centrifugation to prevent antibody loss. Elution was conducted through addition of 5 mL of Elution Buffer (pH 2.8) and centrifugation (1000 g, 1 min, 25 °C). The different eluted fractions were collected and neutralized in tubes with 0.5 mL of 1 M phosphate buffer (pH 8.4). The pH of all buffers and its dilutions must be checked beforehand using a pH meter. Protein concentration was measured in each fraction with a NanoDrop 2000 spectrophotometer, and purity was assessed by SDS-PAGE (see section 3.4, “Protein electrophoresis”).

The average yield was approximately 6 mg of purified ACK2 per 200 mL of antibody-containing supernatant before concentration. This yield, along with the binding capacity of the purification column, should be considered when determining the number of purification rounds required for a given supernatant volume.

Finally, antibody-containing fractions were desalted using spin desalting columns, centrifuged (1000 g, 2 min, 25 °C) and exchanged into sterile PBS, suitable for *in vivo* administration (**Figure 1**). Protein concentration was re-measured, and aliquots were stored at -20 °C. Functional activity was confirmed by *in vitro* competition assay (section 3.6; **Figure 2B**).

### 3.4 Protein electrophoresis

Purified ACK2 samples were mixed with 4X Laemmli sample buffer and 5% DTT. Samples were boiled at 95 °C for 10 min, vortexed briefly every 2 min, and centrifuged (16,000 g, 1 min). Protein electrophoresis was performed on 12% Tris-glycine gels using 2 µg of protein per well and 5 µL of molecular weight standard. Gels were run for 40 min at 200 V, then stained with Quick Coomassie Stain. Protein bands confirmed purity of ACK2 antibody (**Supplementary Figure S**).

### 3.5 Bone marrow and spleen cell isolation

BM cells were obtained by flushing femurs and tibias with flow cytometry buffer (PBS, 5% FBS, 2 mM EDTA) using a 25 G needle and 10 mL syringe until bones appeared white. Suspensions were filtered through a 70 µm strainer. Splenocytes were obtained by cutting spleens into two pieces and injecting 10 mL of flow cytometry buffer with a 25 G needle and 10 mL syringe until they appeared white. The obtained cell suspension was filtered through a 70 µm strainer and remaining spleen pieces were mashed in the same filter using the piston of a 1 mL sterile syringe. BM and spleen filtered suspensions were centrifuged (450 g, 5 min), incubated with 5 mL or 10 mL of lysis buffer respectively (5 min, 37 °C, 5% CO_2_, 95% relative humidity), washed and centrifuged (450 g, 5 min).

### 3.6 ACK2 antibody *in vitro* competing assay

ACK2 presence and functionality were evaluated using a flow cytometry-based competition assay (**Figures 1**, **2**).

For detection in supernatants, 2 x 10^6^ RBC-lysed BM cells from C57BL/6 mice were pre-incubated for 15 min at 4 °C with either ACK2 supernatant or RPMI medium (positive control) (**Figure 2A**).

For functional testing, BM cells were incubated under the same conditions with three dilutions (1/10, 1/100, 1/1000) of purified ACK2 antibody (2 mg/mL) or PBS (positive control) (**Figure 2B**).

Cells were then washed and stained with biotin-labeled lineage (Lin) markers followed by streptavidin-APC to distinguish Lin^+^ (majority) from Lin^–^ (HSPC-containing) fractions. Cells were co-stained with PE–anti-c-Kit (clone ACK2) or BUV395–anti-c-Kit (clone 2B8). Flow cytometric analysis showed that pre-incubation with ACK2 supernatant or purified antibody blocked detection of Lin^–^ c-Kit^+^ HSPCs by the PE-labelled clone ACK2, confirming antibody presence and activity. Clone 2B8, recognizing a non-competing c-Kit epitope, remained capable of detecting HSPCs.

### 3.7 Flow cytometry analysis

Cell suspensions were stained with specific antibody combinations in flow cytometry buffer for 15 min at 4 °C. Fc receptors were blocked prior to incubation using FcBlock (anti-CD16/CD32). Samples were analyzed on an LSRFortessa cytometer, and data were processed with FlowJo v10 software. See **Table 1** for used antibody specifications.

### 3.8 *In vivo* ACK2 administration, antibody clearance, and HSPC detection

**Figure 3:**
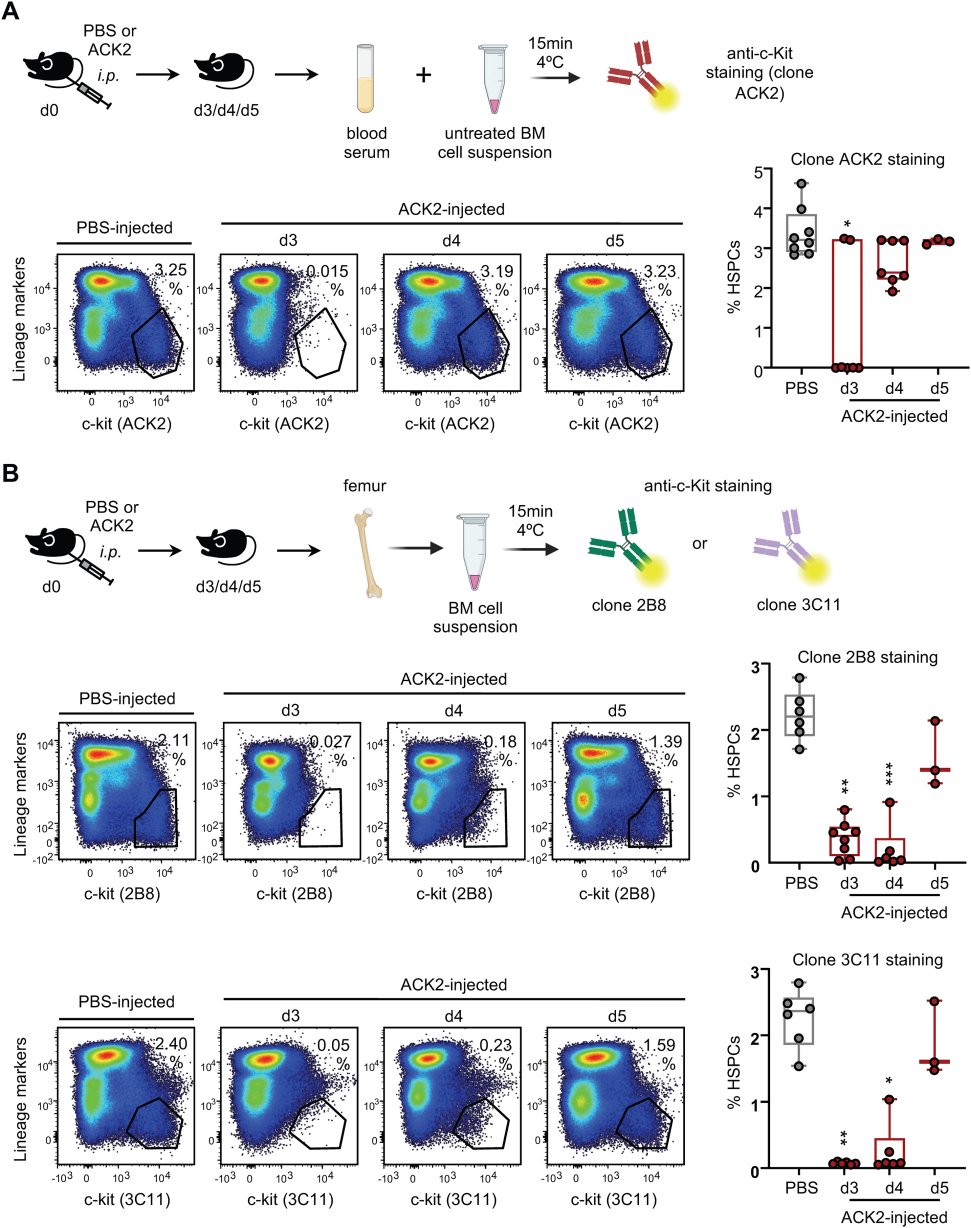
ACK2 antibody clearance from circulation and depletion of BM HSPCs. **(A)** Experimental design: C57BL/6 mice were injected i.p. with PBS or 500 μg of purified ACK2 antibody and peripheral blood was collected at days 3, 4, and 5 post-injection for the detection of ACK2 antibody in serum. Representative dot plots showing competition of serum-ACK2 with fluorescently labeled anti-c-Kit clone ACK2 for binding to Lin^–^ c-Kit^+^ BM cells, and corresponding boxplots showing median cell percentages and IQR with whiskers representing the lowest and highest values. **(B)** Experimental design: C57BL/6 mice were injected i.p. with PBS or 500 μg of purified ACK2 antibody and BM was collected at days 3, 4, and 5 post-injection for the detection of BM HSPCs. Representative dot plots showing Lin^–^ c-Kit^+^ cells labeled with anti-c-Kit clones 2B8 or 3C11 in PBS- and ACK2-injected mice, with boxplots showing median cell percentages and IQR with whiskers representing the lowest and highest values. Statistical significance was determined by non-parametric Kruskal-Wallis test with Dunn’s *post hoc* multiple comparisons (*P < 0.05; **P < 0.01; ***P < 0.001). Dot plots shown are representative of individual samples. Data is a pool of two independent experiments from two independent ACK2 production batches. n = 3–8 mice per condition. Figure partially created in BioRender.com. Guiu, **(A)** (2026).

Purified ACK2 antibody (500 µg in 250 µL PBS/mouse) was administered intraperitoneally (**Figure 3**).

For antibody clearance kinetics, blood from ACK2- or PBS-injected mice was collected from the maxillary vein, centrifuged twice (3000 g, 5 min) to obtain cell-freeserum, and incubated with 2 x 10^6^ RBC-lysed BM cells from untreated mice for 15 min at 4 °C. Cells were fluorescently stained with lineage markers and anti-c-Kit (clone ACK2) and analyzed by flow cytometry (**Figure 3A**).

For HSPC depletion kinetics, RBC-lysed BM cells from ACK2-injected mice were fluorescently stained with lineage markers and anti-c-Kit antibodies (clones 2B8 or 3C11) and analyzed as above (**Figure 3B**). BM from PBS-injected mice served as control.

### 3.9 HSPC purification and adoptive transfer

HSPCs were obtained from DsRed.T3 donors by flushing femurs and tibias with PBS using a 25 G needle and 10 mL syringe until bones appeared white. Suspensions were filtered through a 70 µm strainer, extensively washed and centrifuged (450 g, 5 min). Lin^–^ HSPC-enriched cells were isolated by immunomagnetic depletion using the Lineage Cell Depletion Kit and AutoMACS Pro Separator, according to the manufacturer’s instructions. The Lin^–^ HSPC-enriched fraction was washed, centrifuged, resuspended in PBS, and transplanted intravenously into recipient C57BL/6 mice (1 x 10^6^ Lin^–^ cells in 0.1 mL PBS per mouse).

### 3.10 *C. albicans* infection

*C. albicans* PCA2 (low-virulence, non-germinative strain) or the virulent strain ATCC 26555 were cultured in endotoxin-free YPD medium (1% yeast extract, 2% peptone, 2% dextrose) at 28 °C with gentle agitation until exponential growth (A_600_ = 0.6-0.8). Yeasts were pelleted (7200 g, 5 min), resuspended in sterile water, and incubated for 3 h at 28 °C with shaking. Cultures were then starved at 4 °C for 24 h before use. Yeast cells were quantified by microscopic counting using a Neubauer chamber, washed, and diluted in PBS to the desired concentration.

- To induce trained immunity: 1.5 x 10^6^ PCA2 yeast cells were injected intravenously (0.1 mL) into DsRed.T3 mice. PBS was used as control.
- To assess neutrophil recruitment: 1 x 10^7^ ATCC 26555 yeast cells were injected intraperitoneally (0.2 mL) per mouse.

### 3.11 Peritoneal cavity lavage

Peritoneal cells were isolated as previously described (18). Mice were euthanized by cervical dislocation and placed supine and disinfected with 70% ethanol. A midline skin incision was made from the abdomen to the neck without penetrating the peritoneum, then extended to hind- and forelimbs to expose the cavity. 10 mL of cold flow cytometry buffer were injected into the peritoneal cavity using a 25 G needle. The abdomen was gently massaged to release resident and recruited cells. An incision was made in the peritoneal wall, and the lavage fluid was collected into a 15 mL tube via funnel.

### 3.12 Statistical analysis

All statistical analyses were performed with the use of GraphPad Prism v8.4.2 software. Statistical differences for dual comparisons (ACK2-injected *vs.* PBS-injected or PCA2-infected *vs.* PBS-treated) were determined using two-tailed Mann-Whitney U test. Statistical differences for independent multiple comparisons (PBS-injected *vs.* day 3, 4, 5 post-ACK2-injected) were determined using Kruskal-Wallis test followed by Dunn’s *post-hoc* pairwise comparisons. Data are expressed as median with interquartile range (IQR). Significance was accepted at *P < 0.05, **P < 0.01, and ***P < 0.001 levels.

## 4 Results

### 4.1 Bone marrow HSPC depletion and clearance of ACK2 antibody from circulation are achieved 4 days after ACK2 administration in mice

Previous studies demonstrated that administration of the monoclonal anti-c-Kit antibody (clone ACK2) in Rag2^-/-^ mice induces HSPC ablation, thereby enhancing hematopoietic reconstitution (2). In this study, we aimed to clear BM niches to improve the adoptive transfer of donor HSPCs. To accomplish this, it was necessary to account for the residual ACK2 antibody remaining in circulation, which could otherwise deplete transplanted donor HSPCs.

To determine the *in vivo* kinetics of antibody clearance, we administered 500 µg of ACK2 intraperitoneally and tested serum samples collected at days 3, 4, and 5 post-injection. The presence of circulating ACK2 was assessed by an *in vitro* competing assay: BM cells were incubated with serum samples, then stained with a fluorescently labelled lineage markers cocktail and anti-c-Kit clone ACK2 antibody, and analyzed by flow cytometry (**Figure 3A**). The results showed that ACK2 was detectable in serum on day 3, as HSPCs could not be labelled with the fluorescent ACK2 anti-c-Kit antibody, but was absent by days 4 and 5 (**Figure 3A**).

ACK2 treatment could be inducing a transient depletion of host HSPCs, potentially creating a short time window during which recipient mice might be receptive to donor HSPC transplantation. We therefore sought to define this “transplantation window”, characterized by both the absence of circulating ACK2 and depletion of endogenous HSPCs in C57BL/6 mice. To this end, BM cells were collected at days 3, 4, and 5 after ACK2 administration (**Figure 3B**). HSPCs were identified as Lin^–^ c-Kit^+^ cells using two distinct anti-c-Kit clones, 2B8 and 3C11. The use of clones 2B8 and 3C11 allowed us to distinguish between ACK2 antibody bound to c-Kit and true HSPC depletion, as these antibodies recognize non-competing epitopes. Flow cytometric analysis revealed that HSPCs were undetectable in BM at days 3 and 4 post-injection but had reappeared by day 5 (**Figure 3B**).

Together, these data define day 4 post-ACK2 treatment as the optimal transplantation window, coinciding with maximal endogenous HSPC depletion and complete clearance of circulating antibody.

### 4.2 Enhanced engraftment of HSPCs in the ACK2-based HSPC depletion mouse model

**Figure 4:**
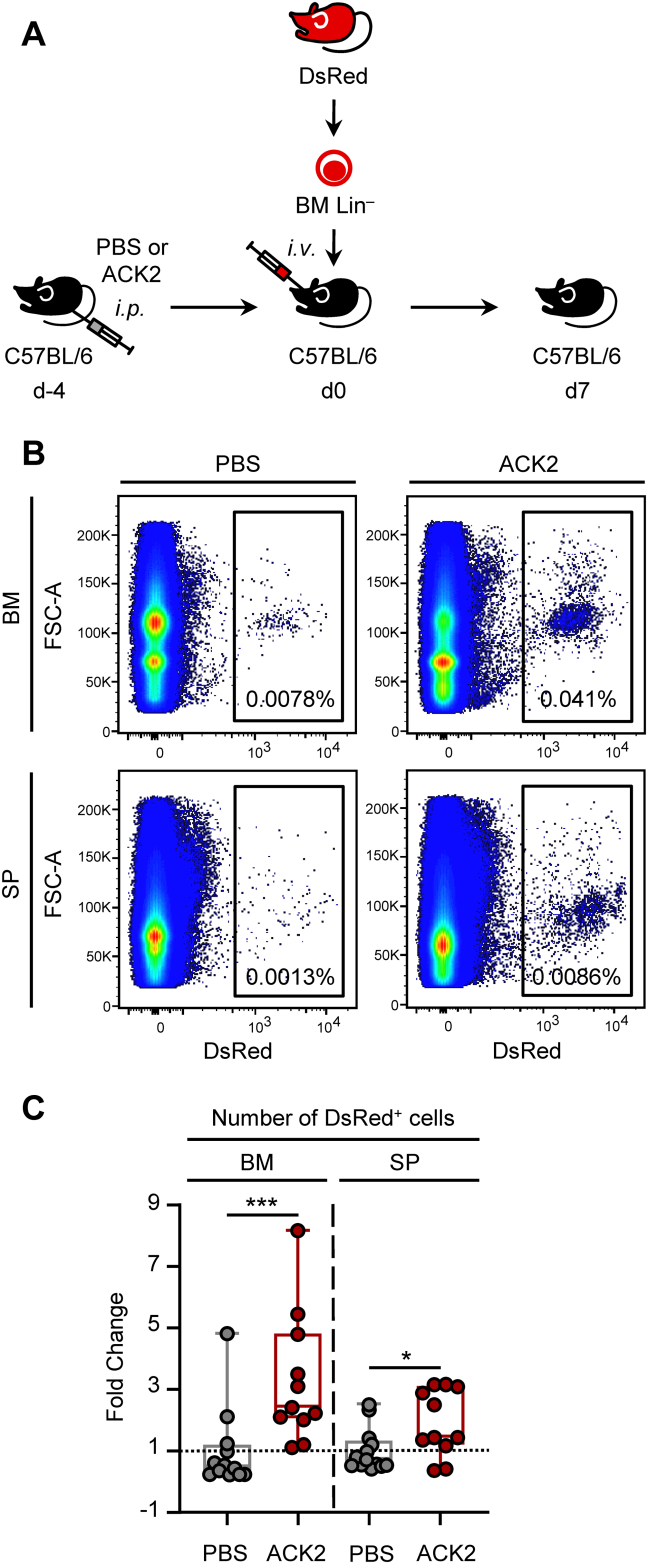
HSPC engraftment in ACK2-conditioned mice. **(A)** Experimental design: C57BL/6 mice were injected i.p. with PBS or 500 μg purified ACK2 antibody 4 days before transplantation. Lin^–^ from DsRed donors were adoptively transferred i.v. into PBS- or ACK2-conditioned recipients, and differentiation was allowed for 7 days. **(B)** Representative dot plots showing the percentages of DsRed^+^ cells in the BM and spleen of PBS- or ACK2-conditioned recipients. **(C)** Fold change in the total number of DsRed^+^ cells, normalized to the mean of PBS controls, in BM and spleen of PBS- or ACK2-conditioned recipients, shown as boxplots showing median cell percentages and IQR with whiskers representing the lowest and highest values. Statistical significance was determined by non-parametric Mann-Whitney U test for dual comparisons (*P < 0.05; ***P < 0.001). Representative dot plots are a concatenated of all samples from one experiment. Data is a pool of three independent experiments from two independent ACK2 production batches. n =11–14 mice per condition.

We next evaluated whether ACK2-mediated depletion of host HSPCs could improve donor engraftment in C57BL/6 mice. For this purpose, recipient C57BL/6 mice were i.p. injected with either 500 µg of purified ACK2 or PBS. Four days later, Lin^–^ HSPC-enriched cells were isolated from the BM of DsRed donor mice and a total of 1 x 10^6^ donor cells were transplanted i.v. into either PBS- or ACK2-conditioned recipients. We have previously determined that functional imprinting and enhanced responses are reliably detected at day 7 post-transplantation (15, 16). Therefore, seven days after transplantation, host BM and spleen were analyzed for the presence of donor-derived DsRed^+^ cells by flow cytometry (**Figure 4A**).

The results demonstrated a significant fold increase in donor cell numbers in ACK2-conditioned mice compared with PBS controls, with a much greater difference observed in BM than in spleen (**Figures 4B, C**). Engraftment was assessed in both tissues because transplanted HSPCs have been reported to localize in BM and spleen (19, 20).

Together, these findings demonstrate that host conditioning with the ACK2 antibody enhances the engraftment efficiency of adoptively transferred donor HSPCs in C57BL/6 mice. Moreover, we verified the optimal transplantation window, a time point when circulating ACK2 antibody is cleared, and BM niches are transiently available for donor cells.

### 4.3 Improved recruitment of neutrophils derived from trained HSPCs to the peritoneal cavity

To exemplify the applicability of the ACK2 antibody-based HSPC depletion for transplantation studies, we designed a specific *in vivo* scenario.

Our group previously demonstrated that HSPCs from mice infected with the low-virulence, non-germinative *C. albicans* strain PCA2 are reprogrammed to generate trained macrophages with enhanced proinflammatory cytokine production, conferring protection against secondary infection (15). More recently, we showed that HSPCs exposed *in vitro* to *C. albicans* give rise to trained neutrophils, characterized not only by elevated cytokine production but also by superior microbicidal activity due to increased mitochondrial ROS (mtROS) generation (16). Beyond cytokine secretion and microbicidal activity, additional neutrophil functions such as recruitment to infection sites are critical for pathogen clearance. Based on this, we hypothesized that *C. albicans*-reprogrammed HSPCs could generate neutrophils with improved recruitment capacity *in vivo*.

**Figure 5:**
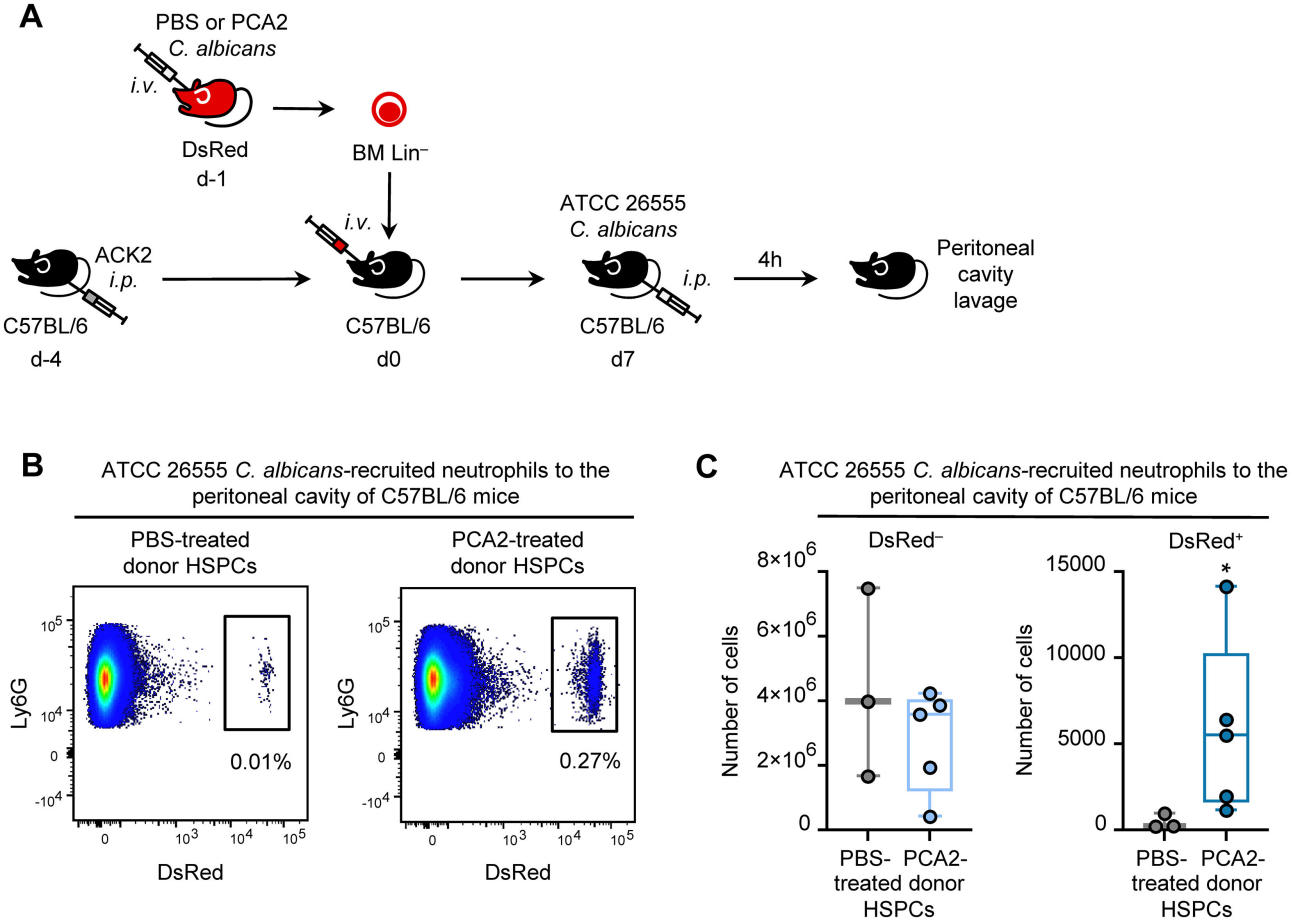
Peritoneal recruitment of neutrophils derived from trained HSPCs in ACK2-conditioned mice. **(A)** Experimental design: C57BL/6 mice were injected i.p. with 500 μg purified ACK2 antibody 4 days before transplantation. One day prior to transplantation, DsRed donor mice were injected i.v. with PBS or 1.5 x 10^6^ yeasts of the non-virulent *C. albicans* strain PCA2. Lin^–^ isolated from DsRed donors were adoptively transferred i.v. into ACK2-conditioned recipients, and differentiation was allowed for 7 days. At day 7, recipient mice were challenged i.p. with 1 x 10^7^ yeasts of the virulent *C. albicans* strain ATCC 26555. Peritoneal cells were collected 4 h post-infection. **(B)** Representative dot plots showing percentages of DsRed^+^ cells among peritoneal neutrophils (CD11b^+^ Ly6G^hi^). **(C)** Numbers of host-derived DsRed– (left) and donor-derived DsRed+ (right) neutrophils recruited to the peritoneal cavity, expressed as boxplots showing median cell percentages and IQR with whiskers representing the lowest and highest values. Statistical significance was determined by non-parametric Mann-Whitney U test (*P < 0.05). Representative dot plots are a concatenated of all samples from one experiment. Data from one representative of two experiments. n = 3–5 mice per condition.

To test this hypothesis, HSPCs isolated from DsRed donor mice either infected for 24 h with PCA2 or treated with PBS were adoptively transferred into C57BL/6 recipients preconditioned with ACK2 antibody (500 µg, i.p.) four days earlier. Seven days post-transplantation, recipient mice were i.p. challenged with the virulent *C. albicans* strain ATCC 26555, and four hours later, peritoneal lavage was performed to recover recruited immune cells (**Figure 5A**). Under steady-state conditions, neutrophils are absent from the peritoneal cavity, and four hours following *C. albicans* challenge is characterized by a selective and robust recruitment of neutrophils.

Flow cytometric analysis of peritoneal cells revealed comparable numbers of host-derived (DsRed^–^) neutrophils (CD11b^+^ Ly6G^hi^) (**Figure 5C**), indicating that the treatment of adoptively transferred HSPCs did not influence host-derived myeloid responses. In contrast, significantly higher numbers of donor-derived (DsRed^+^) neutrophils originating from PCA2-infected HSPCs were recruited to the peritoneal cavity compared with neutrophils from PBS-treated HSPCs (**Figures 5B, C**).

These findings demonstrate that neutrophils derived from *C. albicans*-reprogrammed HSPCs exhibit an enhanced capacity for recruitment to the site of infection during a secondary *C. albicans* challenge. This experimental model exemplifies how antibody-mediated HSPC depletion with ACK2 facilitates efficient adoptive transfer, enabling the detection and functional assessment of donor-derived cells in remote regions of the BM that would otherwise remain undetectable in non-conditioned hosts.

## 5 Discussion

Our study establishes an antibody-based conditioning model for transient HSPC depletion in immunocompetent mice, enabling improved engraftment of adoptively transferred donor progenitors without the toxicity and inflammation associated with irradiation or chemotherapy. We demonstrate that anti-c-Kit ACK2 antibody efficiently depletes BM HSPCs, with maximal depletion and minimal residual antibody at day 4 post-injection, defining an optimal transplantation window. Importantly, this timing was selected according with maximal depletion of endogenous HSPCs and complete clearance of circulating antibody, minimizing the risk of donor progenitor loss. Our results prove that donor HSPCs transplanted at this time show significantly enhanced engraftment, especially in the BM, compared with unconditioned controls. Notably, we obtained consistent results across two independent ACK2 production batches, which strengthens the robustness of our model and confirms its reproducibility.

These findings add to a growing body of literature exploring antibody-based conditioning as an alternative to conventional myeloablation. Classical approaches, including irradiation and chemotherapy, are effective but cause significant morbidity and mortality through inducing broad tissue damage triggering infections, graft *vs*. host disease, infertility, and secondary cancers (1). In contrast, antibody-mediated depletion offers a more targeted and less toxic means of generating niche space for donor progenitors. The pioneering work of Czechowicz et al. showed that ACK2 efficiently depleted HSPCs in Rag2^-/-^ mice, enabling robust engraftment (2). However, subsequent studies revealed that ACK2 alone was insufficient to achieve long-term hematopoietic reconstitution in immunocompetent recipients (5). This apparent discrepancy is not contradictory, as our data confirm, ACK2 induces transient but incomplete depletion, with host HSPCs reappearing as early as day 5 post-injection. This recovery likely reflects the persistence of quiescent long-term hematopoietic stem cells (HSCs), which are resistant to SCF blockade and rapidly repopulate the BM once antibody levels decline.

To achieve durable depletion suitable for clinical translation, several groups have combined ACK2 with complementary strategies. It has been shown that combining ACK2 with CD47 blockade eliminates over 99% of host HSCs, enabling robust multilineage engraftment (6). Conjugation of anti-c-Kit antibodies with toxins such as saporin markedly increases depletion potency (7). The use of 5-azacytidine in combination with anti-CD117 also enhances donor engraftment in immunocompetent mice (8). Moreover, antibody-based conditioning has been reported to enable successful engraftment of haploidentical and fully MHC-mismatched HSCs, while also promoting tolerance to MHC-incompatible organ grafts (21), providing further proof that antibody-based regimens are a promising frontier for both basic and translational hematology research. Within this context, our ACK2 model does not aim to achieve durable hematopoietic reconstitution but rather provides a simpler, transient conditioning strategy optimized for experimental purposes, as a flexible research tool for studying exogenous HSPCs *in vivo*.

The main advantage of our approach is the ability to study exogenous HSPCs *in vivo* under non-inflammatory conditions, where their intrinsic hematopoietic programs remain unperturbed. Inflammatory conditioning can profoundly alter HSPC behavior, either by skewing their differentiation potential or by modifying the programmed functions of their progeny, thus confounding the interpretation of lineage-specific outcomes. This is particularly relevant for research into innate immune memory or trained immunity, a process whereby HSPCs undergo epigenetic and metabolic reprogramming in response to microbial or inflammatory stimuli, generating progeny with enhanced functional potential (11, 12, 22). Our group has contributed to this field by demonstrating that *C. albicans* can reprogram HSPCs to produce trained macrophages with heightened cytokine production (15) and trained neutrophils with enhanced mitochondrial ROS-dependent microbicidal capacity (16). Consistent with these findings, we now show that neutrophils derived from *C. albicans*-exposed HSPCs display superior recruitment to the peritoneal cavity during secondary infection, an effect that may be linked to increased chemokine receptor expression or adhesion molecule upregulation, which merits further investigation. This result complements our previous observations showing that adoptive transfer of *in vitro C. albicans*-stimulated HSPCs into ACK2-conditioned hosts leads to neutrophil progeny with enhanced cytokine production (16). Together, these findings highlight the value of ACK2-based conditioning as an enabling platform to detect and functionally interrogate trained immunity at the progenitor level. Beyond facilitating the engraftment of exogenous HSPCs, this model allows for the detection and functional assessment of their mature progeny *in vivo*, an achievement that is virtually impossible in non-conditioned hosts due to the extremely low engraftment efficiency. Notably, the improved sensitivity of this system enables tracking of donor-derived cells not only within the BM but also across peripheral or anatomically distant sites, thereby expanding the range of analyses that can be performed on their differentiated progeny. Importantly, the absence of confounding inflammatory signals in this system provides a physiological context that more closely resembles steady-state hematopoiesis in the BM.

The applicability of this model could extend beyond facilitating engraftment of exogenous HSPCs. In specific contexts, ACK2 can also be employed to selectively deplete endogenous HSPCs in order to investigate their contribution to disease development or even to host defense mechanisms. For example, our group showed that depletion of c-Kit^+^ progenitors with ACK2 abrogates TLR2 agonist-mediated protection against systemic candidiasis, thereby directly implicating HSPCs in antifungal immunity (23). This model thus provides an opportunity to dissect the role of progenitors in infectious disease, regenerative hematology, cancer or even aging, where understanding the intrinsic properties of HSPCs is essential.

A limitation of this model is that ACK2-mediated depletion is unlikely to eliminate quiescent long-term HSCs. Indeed, we observed HSPC recovery by day 5 post-injection, consistent with previous studies (5). This resistance is likely due to the fact that ACK2 preferentially targets HSPC subsets that depend on SCF/c-Kit signaling for survival, whereas quiescent long-term HSCs, with low proliferative activity and reduced SCF dependency, can persist during the short period in which the antibody remains in circulation and subsequently repopulate BM niches once it is cleared. Consequently, this model is not well suited for interrogating the functional contribution of long-term HSCs. However, the transient depletion provides a short window in which donor HSPCs can engraft and be functionally interrogated before endogenous recovery, which is advantageous for short-term adoptive transfer studies. An intrinsic feature of our non-myeloablative experimental design is the low donor chimerism achieved using ACK2-based conditioning. Nevertheless, despite the low steady-state frequency of HSPCs in these organs, this level of chimerism is sufficient to address short-term functional questions and allows donor-derived progeny to be unambiguously tracked *in vivo*.

In summary, we present a reproducible and accessible model of transient HSPC depletion using ACK2 antibody in immunocompetent mice. This approach enhances adoptive transfer efficiency while avoiding confounding inflammation, thereby enabling precise studies of exogenous HSPCs, their differentiation, and their roles in trained immunity. We propose this method as a valuable addition to the experimental toolbox for immunology, regenerative hematology, and innate immune memory research.

## Data Availability

The original contributions presented in the study are included in the article/**Supplementary Material**. Further inquiries can be directed to the corresponding author/s.
