# Supplementary material for: ACK2 antibody conditioning enhances adoptive transfer of hematopoietic progenitors to study central trained immunity in mice
Source: Front Immunol. 2026 Mar 4;17:1735878. doi: 10.3389/fimmu.2026.1735878 (PMC12996096; doi:10.3389/fimmu.2026.1735878)
Supplement: Supplementary file 1 [file DataSheet1.pdf]

Figure S

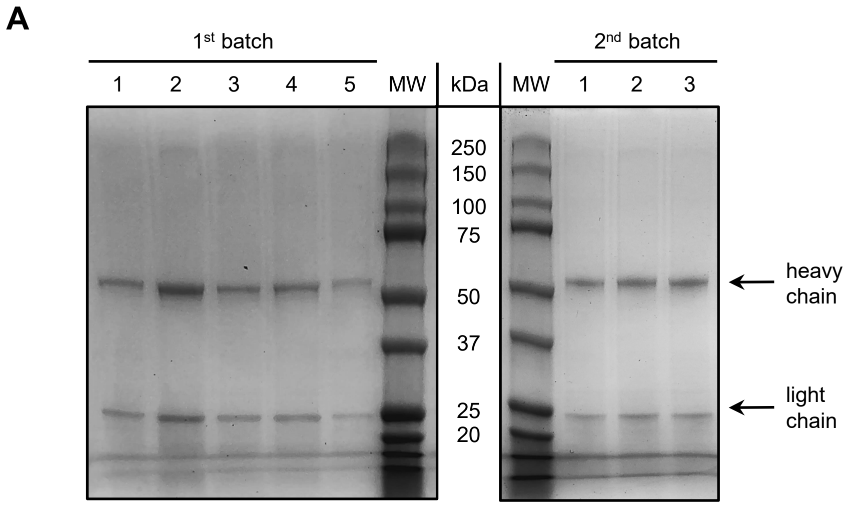

Figure S. Coomassie staining of purified ACK2 antibody batches. Coomassie Brilliant Blue-stained SDS-PAGE gels showing the purity of ACK2 antibody obtained from two independent purification batches. Each lane corresponds to sequential fractions eluted from the affinity chromatography column during the purification process. Both preparations display the expected bands corresponding to the immunoglobulin heavy chain (~50 kDa) and light chain (~25 kDa), consistent with the molecular weight of a mouse IgG2b, κ isotype antibody.
